# Remote sensing of seasonal light use efficiency in temperate bog ecosystems

**DOI:** 10.1038/s41598-017-08102-x

**Published:** 2017-08-17

**Authors:** R. Tortini, N. C. Coops, Z. Nesic, A. Christen, S. C. Lee, T. Hilker

**Affiliations:** 1 Integrated Remote Sensing Studio, Faculty of Forest Resources Management, University of British Columbia, 2424 Main Mall, Vancouver, BC V6T 1Z4 Canada; 2 Faculty of Land and Food Systems, University of British Columbia, 2357 Main Mall, Vancouver, BC V6T 1Z4 Canada; 3 Department of Geography/Atmospheric Science Program, University of British Columbia, 1984 Main Mall, Vancouver, BC V6T 1Z2 Canada; 4 Chair of Environmental Meteorology, Institute of Earth and Environmental Sciences, Faculty of Environment and Natural Resources, Albert-Ludwigs University, Werthmannstr. 10, Freiburg, D-79085 Germany; 5 Department of Geography and Environment, University of Southampton, Highfield Rd, Southampton, SO17 1BJ UK

## Abstract

Despite storing approximately half of the atmosphere’s carbon, estimates of fluxes between wetlands and atmosphere under current and future climates are associated with large uncertainties, and it remains a challenge to determine human impacts on the net greenhouse gas balance of wetlands at the global scale. In this study we demonstrate that the relationship between photochemical reflectance index, derived from high spectral and temporal multi-angular observations, and vegetation light use efficiency was strong (r^2^ = 0.64 and 0.58 at the hotspot and darkspot, respectively), and can be utilized to estimate carbon fluxes from remote at temperate bog ecosystems. These results improve our understanding of the interactions between vegetation physiology and spectral characteristics to understand seasonal magnitudes and variations in light use efficiency, opening new perspectives on the potential of this technique over extensive areas with different landcover.

## Introduction

Although covering just 3% of the Earth’s land surface, wetlands play a crucial role in the global carbon cycle storing more carbon than any other terrestrial ecosystem^1^. Indeed, characterized by moderate primary production but slow decomposition rates, wetland ecosystems store approximately half of the atmosphere’s carbon^2^. However, because of their often remote locations and spatial patchiness, the determination of carbon fluxes between wetlands and atmosphere under current and future climates are associated with large uncertainties. Furthermore, the determination of human impacts on the net greenhouse gas balance of wetlands at the global scale remains challenging^3^.

Near surface remote sensing has been demonstrated to be an effective tool to automatically retrieve quantitative data on physiological processes through the study of the relationship between plant physiological properties and biochemical composition of foliage^4^. This is typically achieved observing narrow spectral wavebands in the 400–2500 nm range with high spectral and spatial resolution optical sensors^5^. Tower-based multi-angular spectroscopy provides improved insights of ecosystem dynamics through linking spectral features of vegetation and carbon fluxes across various spatial platforms and scales^6–13^. This is particularly true in ecosystem models for regional and global estimates of primary production based on light use efficiency^14^ (LUE). The determination of LUE using the photoprotective mechanism in leaves is based on the observation of changes in leaf spectral reflectance, and in particular the epoxidation state of the xanthophyll cycle^15^. The empirical relationship between the photochemical reflectance index^16^ (PRI) and LUE has been demonstrated over a wide range of species^17–24^, proving its potential use for global estimation of LUE using observed spectra. However, the generalization from the leaf level to the canopy, regional, landscape and global scales remains challenging due to the usually relatively small measured reflectance changes^25–27^. In fact, airborne and spaceborne sensors can only provide limited measurements determined by the time of the overpass, whereas the temporal dynamics of plant photosynthesis require the continuous observation of vegetation status under multiple viewing geometries and illumination conditions^28^. Furthermore, canopy level estimates of biophysical parameters from spectral observations are also affected by other variables, such as soil background effects^29^ and sun-observer geometry^30, 31^, making the comparison of measurements taken under different geometries and/or at different times of the day and growing season challenging^32^. In addition, the relationship between PRI and LUE is species dependent and varies with canopy structure, age, Leaf Area Index and possible disturbances^26^. These limitations are further enhanced in the estimation of the fraction of absorbed photosynthetically active radiation (fPAR), subject to bidirectional reflectance and scattering effects^32^ overlapping to the reflectance signal^33^. Previous studies estimated, for example, that grasslands and shrublands showed the lowest mean gross primary production (GPP) and least variability in the continental USA, with mean peak GPP of an order of magnitude lower than deciduous forests^34^. Recent research demonstrated the suitability of LUE for estimation of GPP in peatlands^35^; however, the presence of abundant surface water represents a further limiting factor of spectral observations of chlorophyll signal at wetlands. Among these, global and contiguous multi-angular observations are also affected by the cost associated to instrument networks. The use of low-cost spectro-radiometers is fundamental to overcome this limitation, and the knowledge obtained from these types of studies can then be used to develop models for tower-based vegetation monitoring networks to up-scale reflectance parameters to landscape and global scales. The relationships between canopy reflectance and plant physiological processes at the stand level can be demonstrated using tower-based spectro-radiometers^36, 37^, and low-cost systems such as the third generation Automated Multiangular SPectro-radiometer for Estimation of Canopy reflectance system^13^ (AMSPEC-III) have been developed over recent years. Multi-angular observations acquired at a single location can be used characterize the bidirectional reflectance distribution function^38^ (BRDF) of surface reflectance. This contains information on the structure of vegetated surfaces and shaded parts of the canopy^39, 40^ and facilitates modeling of canopy reflectance independently of the sun-observer geometry, helping to overcome the limitations faced by traditional remote sensing techniques and yield more robust estimates of canopy constituents. For all these reasons, it is crucial to investigate and better understand the relationship between PRI and LUE in complex ecosystems such as wetlands.

**Figure 1:**
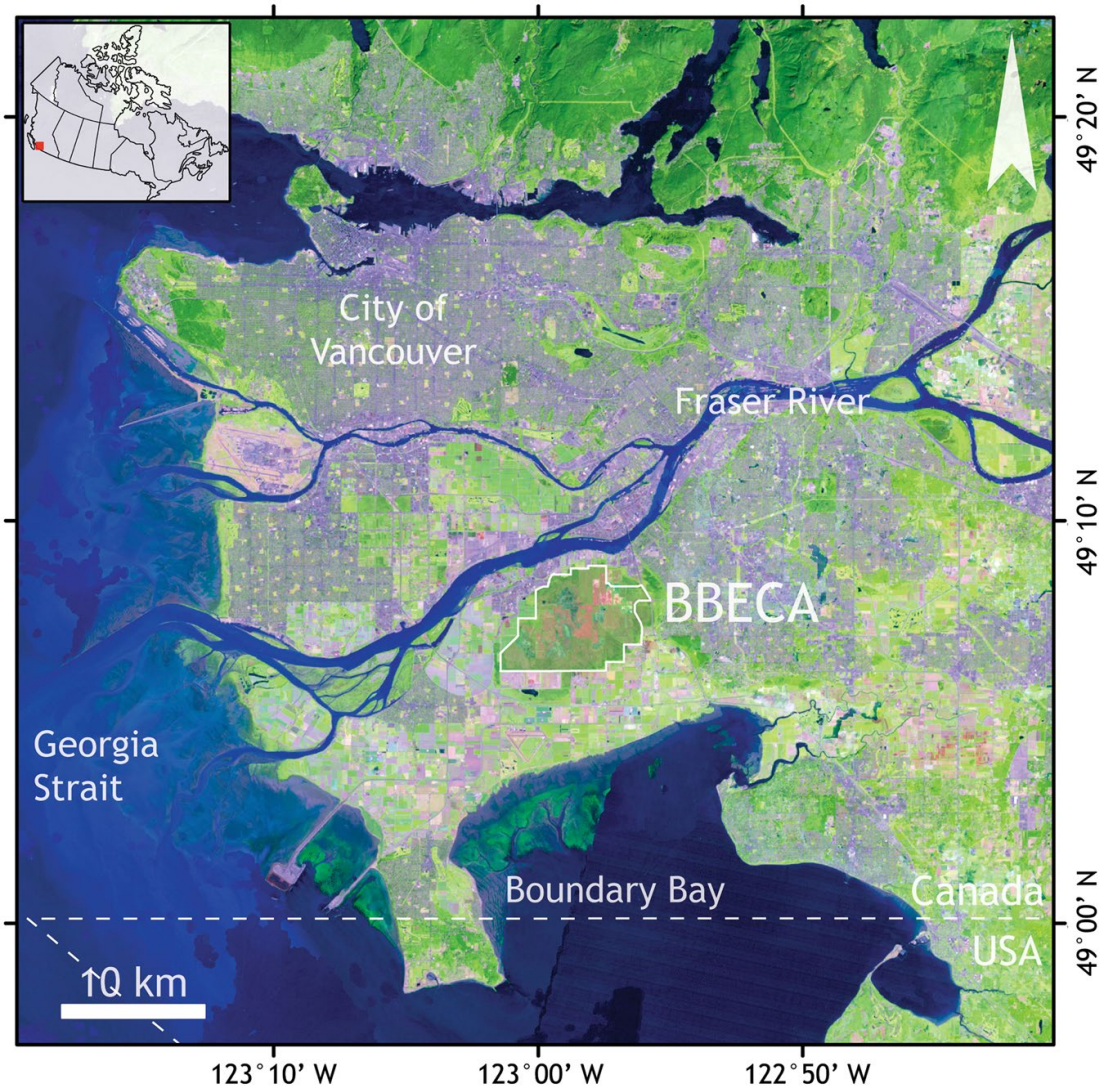
Location of the BBECA within the lower mainland of British Columbia, Canada. Background image from Landsat best available pixel composite^45–50^. Maps generated using ESRI ArcGIS 10.3 (http://www.esri.com/arcgis/).

**Figure 2:**
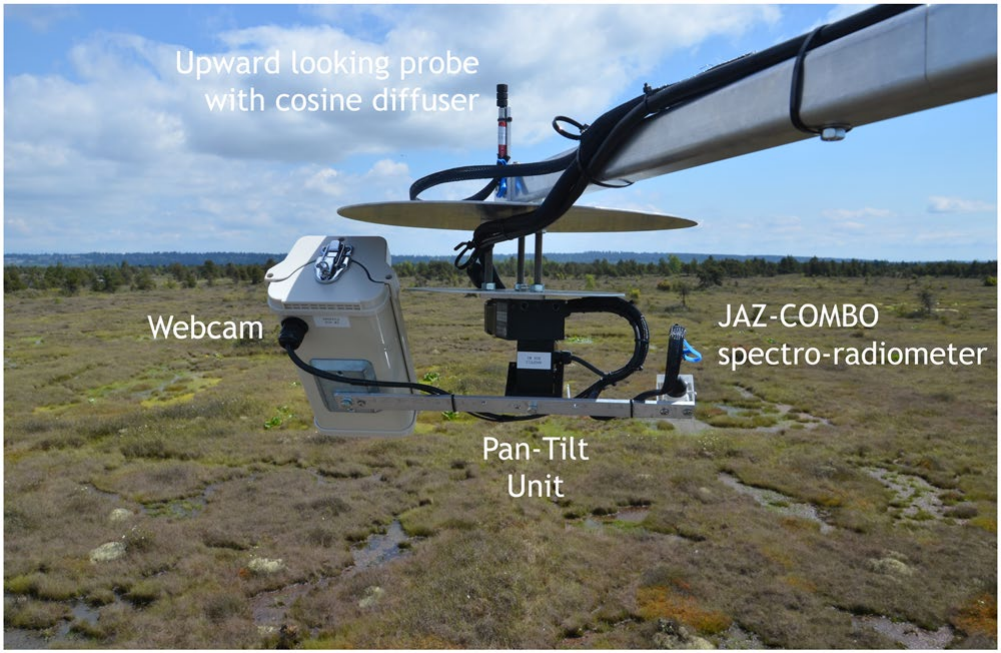
*In-situ* photograph of the AMSPEC-III system taken at the Burns Bog flux tower (Ca-DBB). In the figure the system faces approximately 90°E.

In this work, we assess the relationship between spectral observations under different sun-observer geometry and LUE measurements at a disturbed wetland site. In particular, we utilize the tower-based AMSPEC-III system at an eddy-covariance (EC) flux tower located at the Burns Bog Ecological Conservation Area (BBECA; Figs 1 and 2), a large disturbed, and subsequently restored and rewetted, raised bog ecosystem in the Fraser River Delta on the Pacific Coast of British Columbia, Canada, to establish an empirical relationship between PRI observed using AMSPEC-III and tower-based EC-measured LUE.

## Results

The heterogeneity of the land cover investigated in this study is shown in Fig. 2, including grass, shrubs, and water in addition to anthropogenic elements such as scaffold tower and cabling (not shown in Fig. 2).

**Figure 3:**
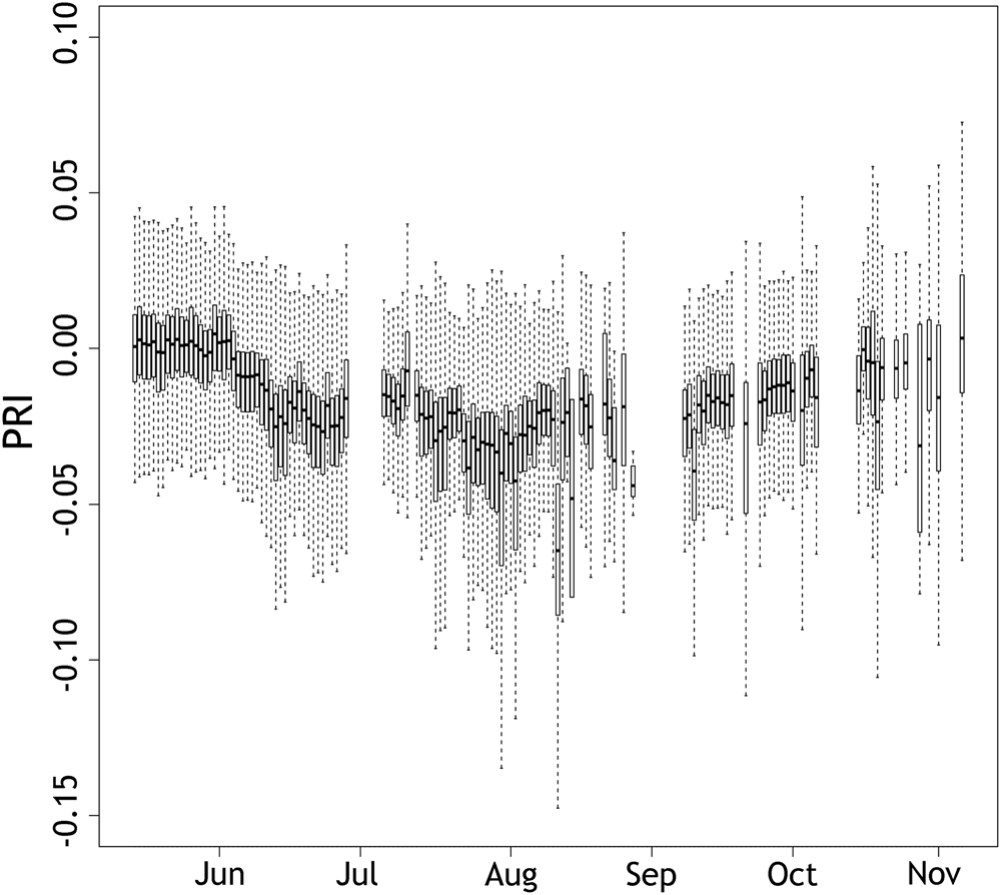
Boxplot of daily PRI distribution at the field site between May and November 2015.

**Figure 4:**
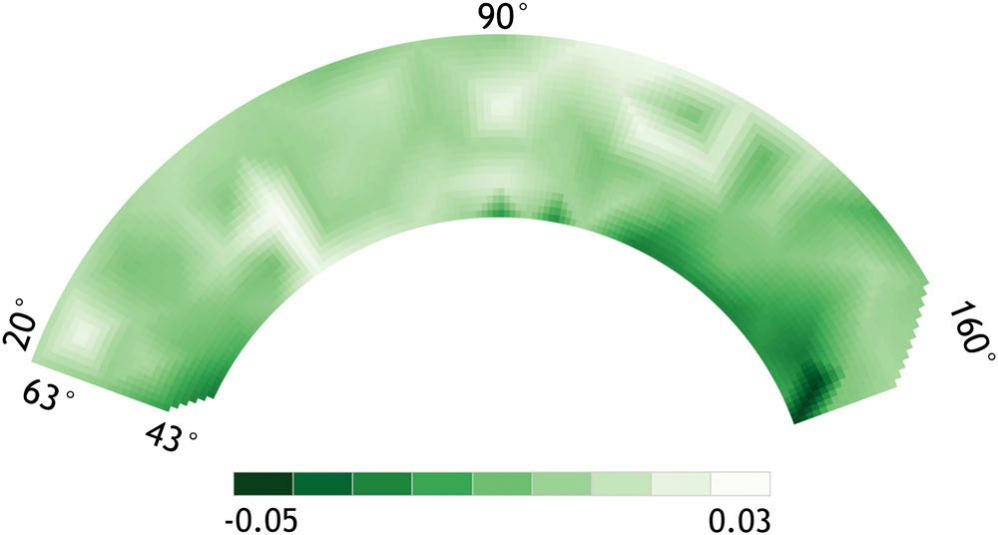
Example of PRI BRDF model for July 31st, 2015.

Although showing a clear seasonal trend (i.e., decrease in PRI values from June to August and increase from September to November), the daily PRI measurements in Fig. 3 as expected are characterized by a high variability due to sun-observer geometry and atmospheric conditions. This spectral variability is a function of the sun-observer geometry, making the BRDF modelling and correction necessary (e.g., Fig. 4). However, for surfaces with heterogeneous landcover such as temperate bog ecosystems, this behavior is also due to high spatial variability associated with the changing land cover types within the field of view.

**Figure 5:**
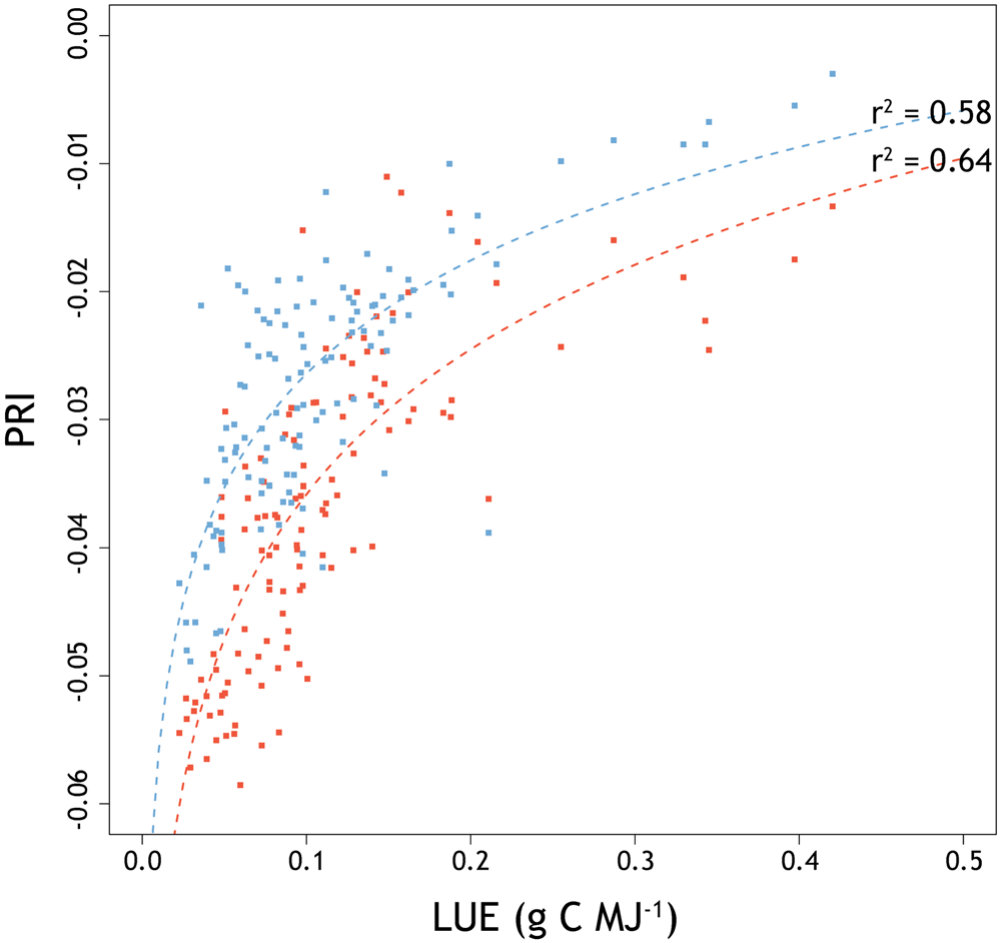
Daily relationship between BRDF-corrected PRI and LUE at the hotspot (red) and darkspot (blue), with logarithmic best fit curve.

In order to calculate LUE throughout the entire season, we modelled the photosynthetically active radiation (PAR) using the measured solar irradiance (*SW*_↓_) at the study site. The relationship between PRI observed at the hotspot (i.e., 20°) and darkspot (i.e., 160°) with the LUE measured from EC is shown in Fig. 5, and shows a significant relationship of r^2^ of 0.64 (*p* < 0.05) and 0.58 (*p* < 0.05), respectively.

**Figure 6:**
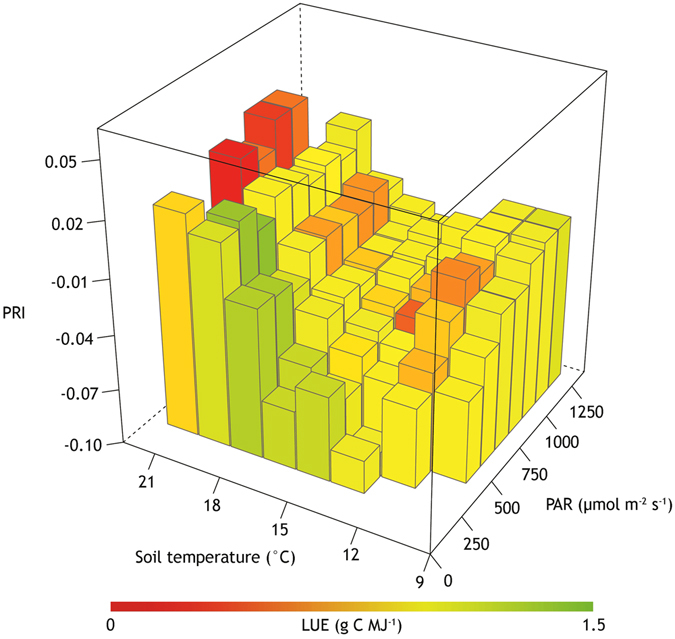
PRI for different PAR and soil temperatures at 5 cm depth. Low PAR values (i.e., diffuse light) generally correspond to lower stress level (i.e., high LUE; green), whereas high temperature to higher stress (i.e., low LUE; red).

The inter-relationship between LUE under different PAR and soil temperature throughout the season is shown in Fig. 6. Low PAR values (i.e., diffuse light) generally correspond to lower stress level (i.e., high LUE; green) and higher PRI, whereas high temperature to lower LUE (red).

## Discussion

A third generation AMSPEC was installed on a flux tower in Burns Bog (Fig. 1), over a restored and rewetted bog ecosystem, in the lower mainland of British Columbia, Canada. AMSPEC-III features a JAZ-COMBO spectro-radiometer (Fig. 2) and, although representing a more affordable option, demonstrated to achieve results in line with previous generations of AMSPEC^13^. The absence of vegetation structure (Fig. 2) combined with the possible presence of surface water made a spatial stratification of the spectral observations necessary. Indeed this heterogeneity significantly affected the measured half hourly PRI, leading to a high daily PRI variability (Fig. 3) throughout the growing season, hence adding further complexity to the already necessary BRDF modelling and correction (Fig. 4). Given the spatial heterogeneity of the site, the hotspot and the darkspot present different landcover; however, both show a good correspondence when compared to LUE (Fig. 5), resulting in r^2^ = 0.64 (*p* < 0.05) and 0.58 (*p* < 0.05), respectively. This is in good accordance with recent studies demonstrating how the relationship between LUE and PRI can be scaled up from foliar to canopy, stand, and even landscape levels^42^.

The inter-relationship between PRI under different PAR and soil temperature (Fig. 6) shows that optimal conditions for vegetation activity in a temperate bog (i.e., high LUE) are characterized by low PAR (<500 µmol m^−2^ s^−1^) and soil temperature comprised between 15 °C and 20 °C. However, PRI also shows moderate activity (i.e., LUE comprised between 1 and 1.5 g C MJ^−1^) for cold sunny days (i.e., soil temperature <15 °C and PAR >1000 µmol m^−2^ s^−1^).

As expected, low PAR values (i.e. diffuse light) generally correspond to lower stress level (i.e., high LUE; green), whereas high temperature to higher stress (i.e., low LUE; red). However, PRI values seem to be affected by the presence of surface water. In fact, for supposedly drier conditions (i.e., high PAR and high soil temperature), PRI remains high also for low LUE values. These results are in good accordance with the conclusions in refs 43 and 44, where PAR and soil temperature were indicated as key environmental factors controlling gas exchanges at this study site in 2014 and 2015, respectively. In particular, high water table and in particular surface ponds seemingly suppress fluxes and affect spectral observations. In fact, water table height at the field site decreases steadily between June and September 2015. Alternatively, due to increased precipitation and reduced evapotranspiration, as a consequence of senescence, water table height rises in September and October. These factors make the study area a CO_2_ sink in spring and summer, whereas CO_2_ fluxes are near zero in fall and winter^44^.

This work demonstrates the AMSPEC-III versatility in ecosystems presenting complex landcover such as bogs. Simultaneous spectral observations of different vegetation types from multiple AMSPEC-III systems can help to better understand the interactions between vegetation physiology and spectral characteristics. In addition, a combined effort of multiple tower-based AMSPEC-III systems in a network could considerably improve the calibration of satellite observations, ultimately leading to improved understanding of changing vegetation spectral features at the global scale. In fact, bogs have mean peak GPP an order of magnitude lower than deciduous forests^34^ and represent a complex landscape and estimating LUE using spectral observations presents a number of challenges, including, for example, spectral variation due not only to sun-observer geometry but also by its heterogeneous landcover.

AMSPEC-III demonstrated to be suitable for carbon cycle analysis in an environment with heterogeneous landcover such as the rewetted bog ecosystem studied here. For these reasons we suggest that, when spatial variability plays a crucial role such as in sensor networks observing a range of different landcover patches over extensive areas (e.g., wetlands located in remote regions), cost effective spectral solutions such as the AMSPEC-III system represent a natural candidate for the implementation of instrument networks at a broader scale.

## Methods

### Study area

Burns Bog is a remnant ombrotrophic raised bog ecosystem expanding for approximately 20 km^2^ located between the south arm of the Fraser River and Boundary Bay in Metro Vancouver, British Columbia (Fig. 1) on a large estuarine delta with chemistry influenced by the nearby marine environment. This, along with its flora supporting distinctive bog vegetation communities and recognized rare and endangered plant and wildlife species, contributes to Burns Bog’s environmental unicity and global significance^45^. Burns Bog contains about 14 km^2^ of disturbed bog ecosystems with previous land uses including peat mining, agriculture or recreation, in addition to 6 km^2^ of relatively undisturbed raised peat bog. While not pristine, the bog has retained enough of its ecological integrity to allow its restoration over time^45^. In 2005, the major remaining bog ecosystem was declared a conservancy area (Burns Bog Ecological Conservancy Area, BBECA) and restoration efforts primarily focus on rewetting by a large-scale ditch-blocking program^46^. To allow the continuous measurements of CO_2_ fluxes using EC, a scaffold tower was installed inside BBECA in summer 2014 on a floating platform (122°59′05.60″W, 49°07′45.59″N, WGS-84, Fluxnet ID Ca-DBB) within a field of 400 × 250 m dimension. This field has been harvested and disturbed between 1957 and 1963, and re-wetted in 2008^43^. The current vegetation is dominated by a white beakrush (*Rhynchospora alba*) - *Sphagnum* ecosystem reaching a height of 30 cm in summer^43^. Sphagnum carpets are discontinuous in this area, with dispersed scrub pine (*Pinus contorta*) and birch (*Betula pendula*) trees surrounding the area observed.

The study area is characterized by standing water ponds intermixed with vegetation for most of the year. Vegetation coverage was almost complete in summer, covering ponds, so the surface was less patchy in summer compared to spring and fall, when standing water ponds were intermixed with vegetation^44^. During the study period (i.e., May 14th to November 6th, 2015) the water table fluctuated between +15 cm (above surface, flooded) and −22 cm (below surface). Summer 2015 was an unusually dry early summer (May to July precipitation: 36 mm, compared to 154 mm in the 30-year climate normal 1981–2010, at Vancouver International Airport). Consequently, the water table in summer 2015 at the study site was lowest compared to the preceding 7 years (start of measurements).

### AMSPEC-III spectral observations

Spectra were measured from May 14th to November 6th, 2015 using an AMSPEC-III system mounted on the flux tower at 4.5 m above reference height (Fig. 2). The AMSPEC-III system features a JAZ-COMBO portable spectro-radiometer (JC; Ocean Optics, Dunedin, FL, USA) with an upward-looking sensor featuring a cosine diffuser to correct sky irradiance for varying solar altitudes. A pan-tilt unit (model PTU-D46–17; Directed Perception, Burlingame, CA, USA) allows the system to record data at various view zenith angles (VZAs) and view azimuth angles (VAAs) from the initial position. In particular, spectra were recorded at VZA ∈ {43°; 53°; 63°} and VZA ∈ {48°; 58°; 68°} every 15 min (half full rotation) and, in order to minimize the influence of the tower apparatus, in this study we focus on 20° ≤ VAA ≤ 160°. In addition to a RGB webcam image (model NetCam SC 5MP; StarDot, Buena Park, CA, USA) automatically acquired and co-registered to the simultaneous spectra, solar irradiance and canopy radiance were recorded simultaneously to the sensors viewing geometry, solar position, time of measurement in order to model the BRDF^36^ and scale the spectral observations. The AMSPEC-III system components are described in ref. 13.

Differences in light sensitivity were corrected by measuring the reflectance of a diffuse reference target (i.e., Labsphere Spectralon^®^) and through a cross-calibration approach of canopy reflectance (*ρ*), defined as the ratio of canopy radiance and solar irradiance^8^:

1 $$\rho =\frac{L\cdot I^{\prime} }{I\cdot L^{\prime} }$$

where *L* is the measured radiance of the canopy sensor, *I* is the simultaneously measured irradiance, *L*′ is the measured radiance of the control surface, and *I*′ is the irradiance at the time *L*′ was measured.

The raw JC spectra were collected with 0.145 nm nominal resolution (2048 channels covering the 200–1100 nm spectral range) and resampled to 3.3 nm full width at half maximum (FWHM) using the arithmetic mean of overlapping bands (*cfr*. ref. 13). The spectra utilized in this work were ultimately configured to 154 wavebands in the 522–809 nm spectral range.

In order to exclude any influence from the platform, the scaffold tower or the wooden boards providing access to the platform itself, the analysis of the spectra was restricted to the region comprised between a view azimuth angle of 20° and 160°.

It is possible to determine vegetation LUE, a key attribute to understand photosynthetic functioning, observing the variations in spectral reflectance resulting from the epoxidation state of the xanthophyll cycle^15^ due to the photoprotective mechanism of leaves. In particular, these variations are displayed as absorption features at 505 and at 531 nm, and can be quantified using the PRI, a normalized index that compares the reflectance at 531 nm to a xanthophyll insensitive wavelength (i.e., 570 nm) defined as^16^:

2 $$PRI=\frac{{\rho }_{531}-{\rho }_{570}}{{\rho }_{531}+{\rho }_{570}}$$

where *ρ*_531_ and *ρ*_570_ are measured reflectance at 531 and 570 nm, respectively.

To reduce the spectral variation associated with changes in land cover we stratified the spectra-derived BRDF models of PRI based on land cover type. This stratification is necessary in order to cluster the main land cover type, under the assumption that similar BRDF kernels should originate from similar land cover types. In order to do so, we selected a clear day (i.e., July 31st, 2015) to fit a BRDF model for VAA comprised between 20° and 160° with a 1° × 1° spatial resolution (Fig. 4). When, within each 1° × 1° cell, PRI was positive for >50% of the observations, the spectra associated with that viewing geometry was excluded from the analysis. The visual comparison of the remaining viewing geometries and the images acquired using the webcam confirmed that this stratification was able to remove standing water ponds, exposed soil and observations which contained sky and non-vegetation components. In addition, in order to have a more homogeneous range of observations, only the central hours of each day (i.e., 1000–1500) were considered.

### Eddy-covariance and climate measurements

Continuous, half-hourly ecosystem fluxes of CO_2_ were measured using an EC system installed on the micrometeorological flux tower. An ultrasonic anemometer-thermometer (model CSAT3; Campbell Scientific, Logan, UT, USA) and an open-path CO_2_/H_2_O infrared gas analyzer (model LI-7500; LI-COR Inc., Lincoln, NE, USA) were operated at 1.8 m height, to measure NEE^47, 48^. Gross Primary Production (GPP) is defined as the difference between Net Ecosystem Exchange (NEE) and daytime ecosystem respiration (R_D_)^49^.

Additionally, climate and environmental conditions were continuously monitored and storead at 5 min intervals. This included soil/water temperatures recorded at the depth of 5 cm using thermocouples (type T), *SW*_↓_ recorded at 4.25 m (CNR-1, 4-component radiometer, Kipp & Zonen, Delft, Netherlands), and incident and reflected PAR (µmol m^−2^ s^−1^) recorded at 4.25 m using a quantum sensor (LI-190; LI-COR Inc., Lincoln, NE, USA).

We calibrated the relationship between PAR and *SW*_↓_ from July 10th, 2015 (i.e., when PAR measurements started) to November 6th, 2015 (i.e., last day of AMSPEC measurements), and obtained the following scaling factor *f*:

3 $$f=1.798\pm 0.026\,\mu mol\,{J}^{-1}$$

**Figure 7:**
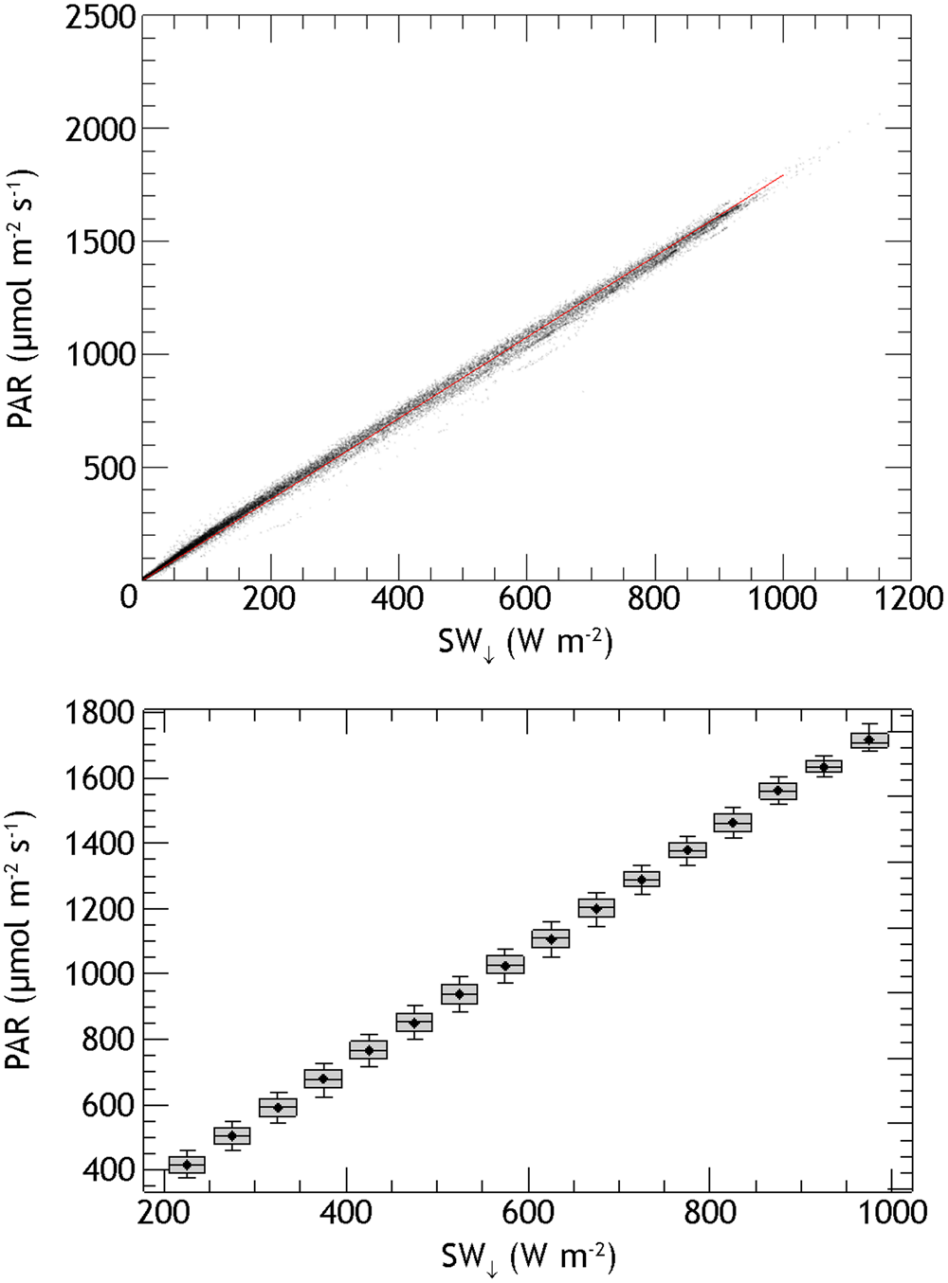
Relationship between PAR and *SW*_↓_: (top) measured PAR and *SW*_↓_ and their linear fit (red line); (bottom) boxplot graph with the binning approach used. The scaling factor *f* is the mean of ratio of the mean PAR and the mean *SW*_↓_ for each bin.

PAR at the field site started being measured on July 9th, 2015. To extend the analysis to the period before the installation of the PAR sensor, we utilized the observed linear relationship between PAR and *SW*_↓_ (in W m^−2^) for all-weather situations (Fig. 7a). More specifically, using a binning approach, and in particular separating 200 ≤ *SW*_↓_ ≤ 1000 into 50 W m^−2^ wide bins, we calculated the ratio of the mean PAR and the mean *SW*_↓_ for each bin (Fig. 7b), and the scaling factor *f* is the mean of all these ratios.

We tested the relationship between the linearly modelled PAR and the actual measured PAR on an independent dataset (i.e., from March 1st to May 1st, 2016) and obtained a RMSE = 22.59 µmol m^−2^ s^−1^ with *r* ^2^ = 0.99 (*p* < 0.05), confirming that this can reliably be used to model PAR before July 10, 2015.

Daily LUE was calculated using half hourly measurements for situations with incident PAR measured at 4.25 m of >10 μmol m^−2^ s^−1^ as follows^14^:

4 $$LUE=\frac{GPP}{PAR\cdot {f}_{PAR}}$$

A detailed description of the EC measurements and the derivation of LUE from EC flux data can be found in ref. 50.
